# Structural basis for the antibody neutralization of *Herpes simplex virus*

**DOI:** 10.1107/S0907444913016776

**Published:** 2013-09-20

**Authors:** Cheng-Chung Lee, Li-Ling Lin, Woan-Eng Chan, Tzu-Ping Ko, Jiann-Shiun Lai, Andrew H.-J. Wang

**Affiliations:** a Institute of Biological Chemistry, Academia Sinica, Taipei 115, Taiwan; b Core Facilities for Protein Structural Analysis, Academia Sinica, Taipei 115, Taiwan; c Development Center for Biotechnology, New Taipei City 221, Taiwan; d Department of Industrial Technology, Ministry of Economic Affairs, Taipei 100, Taiwan; e PhD Program for Translational Medicine, College of Medical Science and Technology, Taipei Medical University, Taipei 110, Taiwan

**Keywords:** *Herpes simplex virus*, glycoprotein D, antibodies, E317

## Abstract

Glycoprotein D (gD) of *Herpes simplex virus* (HSV) binds to a host cell surface receptor, which is required to trigger membrane fusion for virion entry into the host cell. gD has become a validated anti-HSV target for therapeutic antibody development. The highly inhibitory human monoclonal antibody E317 (mAb E317) was previously raised against HSV gD for viral neutralization. To understand the structural basis of antibody neutralization, crystals of the gD ectodomain bound to the E317 Fab domain were obtained. The structure of the complex reveals that E317 interacts with gD mainly through the heavy chain, which covers a large area for epitope recognition on gD, with a flexible N-terminal and C-terminal conformation. The epitope core structure maps to the external surface of gD, corresponding to the binding sites of two receptors, herpesvirus entry mediator (HVEM) and nectin-1, which mediate HSV infection. E317 directly recognizes the gD–nectin-1 interface and occludes the HVEM contact site of gD to block its binding to either receptor. The binding of E317 to gD also prohibits the formation of the N-terminal hairpin of gD for HVEM recognition. The major E317-binding site on gD overlaps with either the nectin-1-binding residues or the neutralizing antigenic sites identified thus far (Tyr38, Asp215, Arg222 and Phe223). The epitopes of gD for E317 binding are highly conserved between two types of human herpesvirus (HSV-1 and HSV-2). This study enables the virus-neutralizing epitopes to be correlated with the receptor-binding regions. The results further strengthen the previously demonstrated therapeutic and diagnostic potential of the E317 antibody.

## Abstract 2

The gD–E317-Fab complex crystal revealed the conformational epitope of human mAb E317 on HSV gD, providing a molecular basis for understanding the viral neutralization mechanism.

## 1. Introduction

Herpes simplex viruses (HSVs) are ubiquitous human pathogens. They are members of the *Alphaherpesvirinae* enveloped dsDNA virus subfamily of the *Herpesviridae*. Two serotypes of HSV, HSV-1 and HSV-2, also known as *Human herpesvirus 1 and 2* (HHV-1 and HHV-2), are neurotropic and are capable of infecting the nervous system and causing neurological diseases such as blindness, meningitis and en­cephalitis. However, these viruses primarily infect oral and epithelial cells, and recurrent infection typically causes oral and genital lesions (Connolly *et al.*, 2011 ▶). HSV-1 and HSV-2 are the most common human viral infections worldwide; they can be transmitted by casual or sexual contact, but HSV-2 is mostly transmitted sexually. These two viruses infect more than 500 million people worldwide, and the newly infected population is estimated to be 23 million each year (Looker *et al.*, 2008 ▶). Therefore, the development of vaccines and novel therapeutic strategies against HSV has become an important research objective.

HSV enters cells through a multistep process *via* the receptor-mediated fusion of the viral envelope with the host cell membrane. The entry-fusion system encompasses a multiprotein complex of virion glycoproteins, gB, gC, gD and gH/gL, and three alternative host receptors, herpesvirus entry mediator (HVEM), nectin-1 and nectin-2 (Karasneh & Shukla, 2011 ▶; Carfí *et al.*, 2001 ▶; Gianni *et al.*, 2009 ▶; Atanasiu *et al.*, 2007 ▶; Geraghty *et al.*, 1998 ▶). gB or gC interacts with 3-*O*-sulfonated heparan sulfate (3-OS HS) on the host cell surface during viral attachment, which facilitates the interaction of gD with its receptors to trigger membrane fusion (Tiwari *et al.*, 2007 ▶). The crystal structures of gD–HVEM (PDB entry 1jma), gD–nectin-1 (PDB entry 3sku), gB (PDB entry 2gum) and gH–gL (PDB entry 3m1c) have been determined (Carfí *et al.*, 2001 ▶; Di Giovine *et al.*, 2011 ▶; Heldwein *et al.*, 2006 ▶; Chowdary *et al.*, 2010 ▶). The conformational changes of gD upon receptor binding initiate binding of the heterodimer gH/gL to gB, thereby activating the fusogenic activity (Atanasiu *et al.*, 2010 ▶). The binding of HSV gD to one of its receptors (HVEM and nectins) causes it to adopt an open conformation and triggers viral fusion. The C-terminus of the gD ectodomain can bind to its N-terminal region and mask the receptor-binding site, resulting in an autoinhibitory closed conformation (Fusco *et al.*, 2005 ▶; Krummenacher *et al.*, 2005 ▶). In the gD–receptor crystals, the gD N-terminus forms a hairpin structure that allows HVEM receptor binding, whereas the nectin-1 binding site encompasses an area that covers both the N-terminal and C-terminal extensions of gD (Carfí *et al.*, 2001 ▶; Di Giovine *et al.*, 2011 ▶). These two cellular receptors have different binding sites on gD.

Several vaccine candidates have been shown to induce a systemic immune response, but they were unable to provide sufficient protection during HSV infection (Looker *et al.*, 2008 ▶). In contrast, neutralizing monoclonal antibodies against gD that block receptor binding have been demonstrated to be an effective mechanism for anti-HSV treatment (Nicola *et al.*, 1998 ▶). To prevent and treat HSV infection, human mAb E317 was generated from a phage-display library of human single-chain variable fragment (scFv) antibodies (Lai & Chan, 2012 ▶). The scFv E317 significantly inhibits HSV-1 (strain KOS) and HSV-2 (strain 186) replication in Vero cells, with IC_50_ values of 5.65 and 3.6 n*M*, respectively. Protection experiments with HSV-infected SCID mice also indicated its efficient inhibition of HSV reproduction. It has been demonstrated that scFv E317 binds to a conformational epitope on gD through immunoprecipitation assays (Lai & Chan, 2012 ▶; unpublished results), but the epitope determinants and neutralization mechanism are not clearly understood.

Here, to understand the neutralization mechanism by characterizing the interactions between gD and antibody E317, the crystal structure of HSV-2 gD in complex with the Fab domain of E317 was determined. The epitope conformation and the detailed interactions clearly show how monoclonal antibody E317 blocks glycoprotein–cellular receptor interactions.

## 2. Experimental procedures

### 2.1. Constructs

The plasmid constructs for mAb E317 (pTCAE8-E317) have been described previously (Anderson *et al.*, 1998 ▶). For HSV-1 gD and HSV-2 gD construction, cDNAs from the gD genes of the HSV-1 KOS strain and HSV-2 strain 186 were used as the DNA templates for constructing plasmids for the C-­terminally truncated gD (residues 1–275), which included a C-terminal His tag, termed HSV-1 gD275 and HSV-2 gD275, respectively. The DNA fragments of HSV-1 gD275 were generated using PCR amplification with primers 5′-CCCTCGAGGCCACCATGGGGCGTTTGACCTCCGG-3′ (forward) and 5′-GACAAGCGGCCGCTA**ATGATGATGATGATGA** **TG**CTCGGGGTCTTCCGGGGCG-3′ (reverse; the sequence encoding the C-terminal His tag is shown in bold), and the DNA fragments of HSV-2 gD275 were amplified with primers 5′-CCCTCGAGGCCACCATGGGGCGTTTGACCTCCGG-3′ (forward) and 5′-GACAAGCGGCCGCTA**ATGATGATG** **ATGATGATG**CTCGGGGTCTTCCGGAACG-3′ (reverse). Both of them were cloned into a pLPCX vector for protein expression.

### 2.2. Production and purification of anti-HSV E317 monoclonal antibody

PER.C6 parental cell lines were thawed and subcultured in a shake flask for two weeks. Electroporation was performed with 8 µg linearized plasmid (pTCAE8-E317) and 6 × 10^6^ viable cells (vc) in 400 µl proprietary basal medium using a Bio-Rad Gene Pulser Xcell. The cells were rested at room temperature for 5 min and the mixture was then transferred to 10.6 ml pre-warmed (309.5 K) basal medium. Six transfection reactions were pooled and cultured in two 15 cm culture dishes in a humidified 309.5 K incubator with 5% CO_2_. After 48 h, the spun cell pellet was re-seeded at a cell density of 5 × 10^5^ vc ml^−1^ in basal medium containing 125 µg ml^−1^ G418. Cells were subcultured twice a week at a density of 3 × 10^5^ vc ml^−1^ with complete fresh medium. When the cell-pool viability reached 50%, the cells were transferred to shake flasks at a density of 5 × 10^5^ vc ml^−1^ at 120 rev min^−1^. When the cell viability recovered to 85% and the cell-doubling time remained consistent after several passages, stable pool cells were frozen and stored in a banked cell in liquid nitrogen.

The fed-batch culture condition of the 20 l bioreactor (Sartorius C bioreactor) started with stable pool cells at an initial cell density of 5 × 10^5^ vc ml^−1^ with viability greater than 90% in 16 l basal medium. The bioreactor settings were as follows: 309.5 K, stir speed of 70–120 rev min^−1^, ring sparger, no lower limit control of pH and 50% air saturation of dissolved oxygen (DO) set point. The harvesting procedure was performed at 50–70% viability by centrifugation (9800 rev min^−1^, 40 min, 277 K) to remove cells. The harvested supernatant was loaded onto a protein A column (GE Healthcare) and washed with PBS; mAb E317 was eluted with 0.1 *M* citric acid pH 4.0.

### 2.3. HSV gD production and purification

HSV-1 gD275 and HSV-2 gD275 have similar expression and purification conditions. Both proteins were expressed in human 293 FreeStyle cells (Invitrogen) by transient trans­fection and the culture supernatants were collected for purification. The His-tagged gD proteins were purified using a nickel–nitrilotriacetic acid column and eluted with 50 m*M* NaH_2_PO_4_, 1 *M* NaCl, 250 m*M* imidazole pH 8.0. Protein solutions were dialyzed against 50 m*M* Tris pH 8.0 and concentrated to 10 mg ml^−1^.

### 2.4. Preparation of E317-Fab and the gD–E317-Fab complex

E317-Fab fragments were prepared by limited digestion with papain (Merck). 1 mg purified mAb E317 (IgG1,κ) was digested with 0.05 mg papain at 303 K for 2 h in a buffer solution consisting of 20 m*M* cysteine, 1 m*M* EDTA, 100 m*M* sodium acetate pH 5.5 and the reaction was terminated by the addition of iodoacetic acid (Sigma) to a final concentration of 70 m*M*. The digested E317-Fab fragment was purified by application onto a protein A column (GE Healthcare) and the unbound Fab fragment was collected in the flowthrough. The Fab fragment was then processed to a final concentration of 10 mg ml^−1^ in 50 m*M* Tris buffer pH 8.0.

To obtain the gD–E317-Fab complex, purified HSV-2 gD and E317-Fab were pre-mixed in a 1:1 molar ratio at 277 K overnight. The mixture was loaded onto a gel-filtration column (Superdex 200 prep-grade XK16/70; GE Healthcare) and the protein complex was eluted at a flow rate of 0.3 ml min^−1^ at 277 K in 50 m*M* Tris buffer pH 8.0. The OD_280_ was monitored for the eluted protein complex. The elution volume corresponding to the gD–E317-Fab complex was then selected for analytical ultracentrifugation and homogeneity analyses.

### 2.5. Analytical ultracentrifugation

Sedimentation-velocity (SV) experiments were performed at 129 000*g* (40 000 rev min^−1^) using a four-hole An-60Ti rotor at 293 K in a Beckman Optima XL-I analytical ultracentrifuge equipped with absorbance optics. The purified HSV-1 gD, HSV-2 gD, E317-Fab and gD–E317-Fab complex samples collected from the gel-filtration column were diluted to a final concentration of 0.3 mg ml^−1^ in 50 m*M* Tris buffer pH 8.0. Standard 12 mm aluminium double-sector centrepieces were filled with protein solution and the reference cell contained blank buffer. Quartz windows were used with absorbance optics (OD_280_) in a continuous mode without averaging. No time interval was set between scans. Data were analyzed with the *c*(*s*) distribution calculated from the Lamm equation using *SEDFIT* v.12.1b (http://analyticalultracentrifugation.com). *SEDNTERP* (http://www.jphilo.mailway.com) was used to estimate the protein partial specific volume (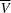), buffer density (1.0 g ml^−1^) and buffer viscosity (0.001 Pa s) at 293 K. HSV-1 gD and HSV-2 gD with 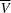 values of 0.74 ml g^−1^ and E317-Fab with a 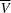 value of 0.73 ml g^−1^ were used to predict the sedimentation coefficient (*S*). The 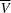 values of the HSV-­1 gD–E317-Fab and the HSV-2 gD–E317-Fab complexes were both 0.73 ml g^−1^.

### 2.6. Crystallization and data collection

Crystals of E317-Fab and the gD–E317-Fab complex were grown by mixing 1 µl protein solution with 1 µl reservoir solution using the sitting-drop vapour-diffusion method at 293 K. The E317-Fab crystals were obtained in a reservoir solution consisting of 25%(*w*/*v*) PEG 4000, 10 m*M* calcium chloride dihydrate, 100 m*M* sodium citrate tribasic dihydrate pH 5.8. The gD–E317-Fab complex crystals were grown from a reservoir solution consisting of 30%(*v*/*v*) PEG 550 MME, 0.1 *M* Na HEPES pH 8.2. All crystals were flash-cooled and the diffraction patterns were recorded at cryogenic temperatures. The diffraction data of E317-Fab crystals were collected at a wavelength of 1.54 Å using a Rigaku FR-E+ SuperBright generator equipped with an R-AXIS HTC image-plate detector. Data for the gD–E317-Fab complex crystal were collected at a wavelength of 0.90 Å on beamline BL44XU of the SPring-8 synchrotron in Japan using an MX-225 CCD detector. Diffraction data were processed and scaled to 2.3 Å resolution using *HKL*-2000 (Otwinowski & Minor, 1997 ▶).

### 2.7. Structure determination and refinement

The E317-Fab crystal structure was determined by molecular replacement using *MOLREP* from the *CCP*4 suite (Winn *et al.*, 2011 ▶) with the BHA10 IgG_1_ Fab fragment (PDB entry 3hc0; Jordan *et al.*, 2009 ▶) as a search model. The E317-Fab crystals belonged to space group *P*2_1_2_1_2_1_, with two Fab molecules in an asymmetric unit.

The gD–E317-Fab complex crystal structure was solved using *MOLREP* with the refined E317-Fab crystal structure and the gD of HSV-1 (PDB entry 1l2g; Carfí *et al.*, 2001 ▶) as search models. The gD–E317-Fab complex crystals also belonged to space group *P*2_1_2_1_2_1_, with one Fab molecule and one gD molecule in the asymmetric unit. Throughout the refinement, a randomly selected 5% of the data were set aside for cross-validation by the *R*_free_ value. Manual modifications of the models were performed using *XtalView* (McRee, 1999 ▶) and *Coot* (Emsley & Cowtan, 2004 ▶). Difference Fourier (*F*_o_ − *F*_c_) maps were calculated to locate the solvent molecules.

The Fab crystal structure was refined for individual atomic positions and temperature factors, and the model yielded *R*_work_ and *R*_free_ values of 20.8 and 26.6%, respectively, using *Crystallography & NMR System* (*CNS*) v.1.2 (Brünger *et al.*, 1998 ▶). The crystal structure of the gD–E317-Fab complex was refined using *REFMAC*5 (Murshudov *et al.*, 2011 ▶), including individual isotropic *B*-­factor refinement and TLS refinement (Winn *et al.*, 2001 ▶), from which *R*_work_ and *R*_free_ values of 20.3 and 25.5%, respectively, were obtained. Data-collection and final model statistics are shown in Table 1 ▶. The molecular figures were produced using *UCSF Chimera* (Pettersen *et al.*, 2004 ▶). The atomic coordinates and structure factors of the crystal structures of E317-Fab and the gD–E317-Fab complex have been deposited in the Protein Data Bank with accession codes 3w9d and 3w9e, respectively.

## 3. Results

### 3.1. Crystal structures

**Figure 1:**
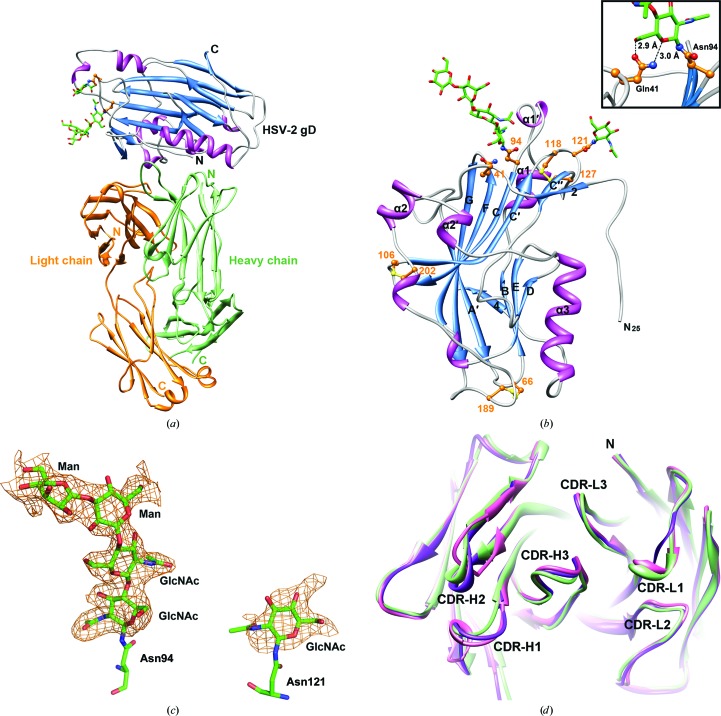
Crystal structure of HSV-2 gD–E317-Fab. (*a*) Ribbon diagram of the HSV-2 gD–E317-Fab complex. gD is coloured blue and magenta and the N-linked oligosaccharides are shown as sticks. The heavy and light chains of E317-Fab are shown in green and orange, respectively. (*b*) Ribbon representation of HSV-2 gD (residues 25–254). Disulfide bonds, two glycosylation sites (Asn94 and Asn121) and the secondary-structure elements are labelled (Carfí *et al.*, 2001 ▶). The inset shows the Asn94-linked *N*-acetylglucosamine (GlcNAc) bound to Gln41. (*c*) OMIT maps of glycans. *F*_o_ − *F*_c_ OMIT maps (orange) were calculated for the two N-linked oligosaccharides and contoured at the 2.0σ level. The glycans and glycan-binding residues are shown as stick models. (*d*) Comparison of E317-Fab. E317-Fab bound to HSV-2 gD (shown in green) and E317-Fab in its unbound form (pink and magenta) are superimposed in the top view. The N-termini and CDRs are indicated.

To elucidate the structural basis by which E317 recognizes the gD antigens, the Fab fragment of mAb E317 and a C-terminally truncated HSV-2 gD construct (residues 1–275) were prepared and purified for crystal structure determination. Firstly, we crystallized the E317-Fab fragment, which contains the heavy chain (residues 1–222) and the light chain (residues 1–214). Next, the crystal structure of HSV-2 gD bound to E317-Fab, including residues 25–254 of HSV-2 gD, residues 1–211 of the light chain and residues 1–136, 142–197 and 201–222 of the E317-Fab heavy chain, was determined (Table 1 ▶). Regions 198–200 and 137–141 in the CH_1_ region of the heavy chain are disordered (Fig. 1 ▶ *a*). Notably, two Asn residues glycosylated with a tetrasaccharide and a monosaccharide attached to the ND2 atoms of Asn94 and Asn121, respectively, were observed (Figs. 1 ▶ *b* and 1 ▶ *c*).

### 3.2. Fab structures

**Figure 2:**
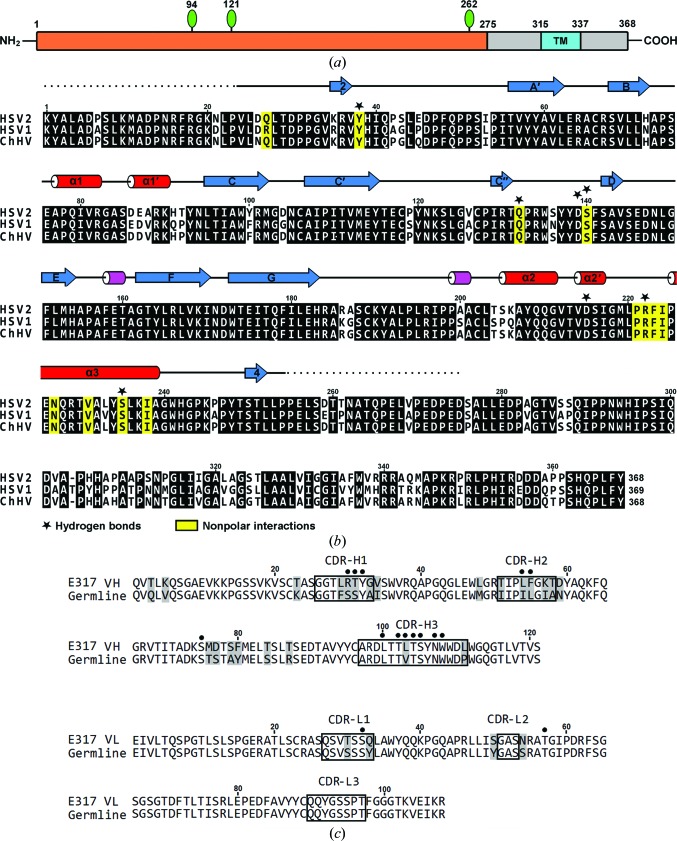
Sequence alignment. (*a*) Schematic representation of HSV-2 gD. The constructed regions (residues 1–275) of gD in this study are represented in orange. N-linked oligosaccharides are shown as green lollipops and a predicted transmembrane (TM) region is highlighted in cyan. (*b*) gD protein sequence alignment between herpesviruses. The gD sequence from *Herpes simplex virus type 2* (NCBI accession No. AAW23130) is aligned with those of the gD proteins of *Herpes simplex virus type 1* (AAA19631) and *Chimpanzee alpha-1 herpesvirus* (BAE47060). Residues in α-helices, β-strands and disordered loops are shown as red cylinders, blue arrows and dotted lines, respectively. The residues involved in the antibody interaction in the gD–E317 complex are marked with yellow boxes (nonpolar interactions) and black stars (hydrogen bonding). (*c*) E317 sequence alignment. The amino-acid sequence of the V and J regions of E317 was aligned with the closest homologous germline suggested by *IMGT/V-QUEST* and junction analysis (Lefranc *et al.*, 2012 ▶). The positions of the CDRs according to the IMGT nomenclature are indicated, whereas somatic mutations are shaded. The residues involved in the antigen interaction in the gD–E317 complex are marked with black dots.

E317-Fab displays the typical immunoglobulin fold. The complementarity-determining re­gions (CDRs; Fig. 2 ▶ *c*) are as defined in the international ImMunoGeneTics information system (IMGT; Lefranc *et al.*, 2009 ▶). In this case, the light-chain CDR loops are composed of residues 27–33 (CDR-L1), 51–53 (CDR-L2) and 90–97 (CDR-L3), and the CDR loops of the heavy chain are composed of residues 26–33 (CDR-H1), 51–58 (CDR-H2) and 97–111 (CDR-H3). See Supplementary Fig. S1^1^ for alternative numbering based on predictions by *abYsis* (http://www.bioinf.org.uk/abysis). In our structure, the CDR loops of CDR-H1, CDR-H2 and CDR-L1 show conformational variations in two independent Fab molecules. In Fig. 1 ▶(*d*) the conformations of the bound and unbound CDR structures are compared, in which the root-mean-square deviations (r.m.s.d.s) of 18 pairs of C^α^ atoms in the CDRs are calculated to be 0.01–1.10 Å (Supplementary Table S1), indicating limited structural differences.

The two independent Fab structures (heterodimers HL and H′L′) are nearly identical, with an r.m.s.d. of 0.40 Å for 209 C^α^ atoms, but show slightly different conformations of CDR-H1, CDR-H2 and CDR-L1 (Fig. 1 ▶ *d*). The CDR-H1 and CDR-L1 regions have helix conformations in heterodimer HL but form loops in heterodimer H′L′, with r.m.s.d. values of 0.90 Å (H1) and 0.78 Å (L1). In contrast, the CDR-H2 region has a loop conformation in heterodimer HL but forms a helix in heterodimer H′L′, with an r.m.s.d. of 1.10 Å between them. The loop conformations of CDR-H3, CDR-L2 and CDR-L3 in the gD-bound E317-Fab structure remain similar to those in the two independent Fab structures. The helix conformations of CDR-H1 and CDR-L1 are similar to the unbound heterodimer HL, with r.m.s.d. values of 0.40 and 0.19 Å, respectively. The CDR-H2 region forms a helix conformation which is similar in both heterodimers HL and H′L′, with an r.m.s.d. of 0.29 Å.

### 3.3. HSV-2 gD structure and glycosylation

HSV-2 gD contains 368 residues, with a transmembrane segment from residues 315 to 337 and three N-linked glycosyl­ation sites at residues 94, 121 and 262 (Fig. 2 ▶ *a*), as predicted by both the *SOSUI* (Hirokawa *et al.*, 1998 ▶) and the *NetNGlyc* programs (http://www.cbs.dtu.dk/services/NetNGlyc).

In the current structure of E317-Fab-bound HSV-2 gD, the N-terminus starts at residue 25 and the C-terminus ends at residue 254. The first 24 and last 21 residues, including the His tag, were highly flexible and were not observed. As shown in Fig. 1 ▶(*b*), the overall structure of HSV-2 gD presented here is similar to that of HSV-1 gD (PDB entry 2c36; Krummenacher *et al.*, 2005 ▶), with an r.m.s.d. value of 0.50 Å for 225 C^α^ atoms when the two structures are superimposed. The core structure folds into a nine-stranded (*A*, *B*, *C*, *C*′, *C*′′, *D*, *E*, *F* and *G*) V-­type Ig-like domain plus three short β-strands (2, 3 and 4). Five α-helices (α1, α1′, α2, α2′ and α3) and two 3_10_-helices are inserted as follows: α1 and α1′ between strands *B* and *C* and α2, α2′ and α3 plus one 3_10_-helix between strands *G* and 4. They are connected to the core domain by two disulfide bridges: Cys66–Cys189 and Cys106–Cys202. Two cysteines (Cys118 and Cys127) in the *C*′*C*′′ loop form the third disulfide bond and there is one N-linked glycosylation motif between them.

Two oligosaccharides attached to Asn94 and Asn121 have been identified in this study (Fig. 1 ▶ *c*). The glycosylation at residue Asn262 remained uncertain because the C-termini were disordered. The glycan structure at Asn94 has a four-sugar structure, Manα1–3Manβ1–4GlcNAcβ1–4GlcNAcβ-Asn94, in which the first GlcNAc is hydrogen bonded to the side chain of Gln41 to stabilize the loop between strands 2 and *A*′. Only the first GlcNAc residue can be observed at Asn121 and it does not show any interaction with other amino acids (Fig. 1 ▶ *b*).

### 3.4. The HSV-2 gD–E317-Fab interface

**Figure 3:**
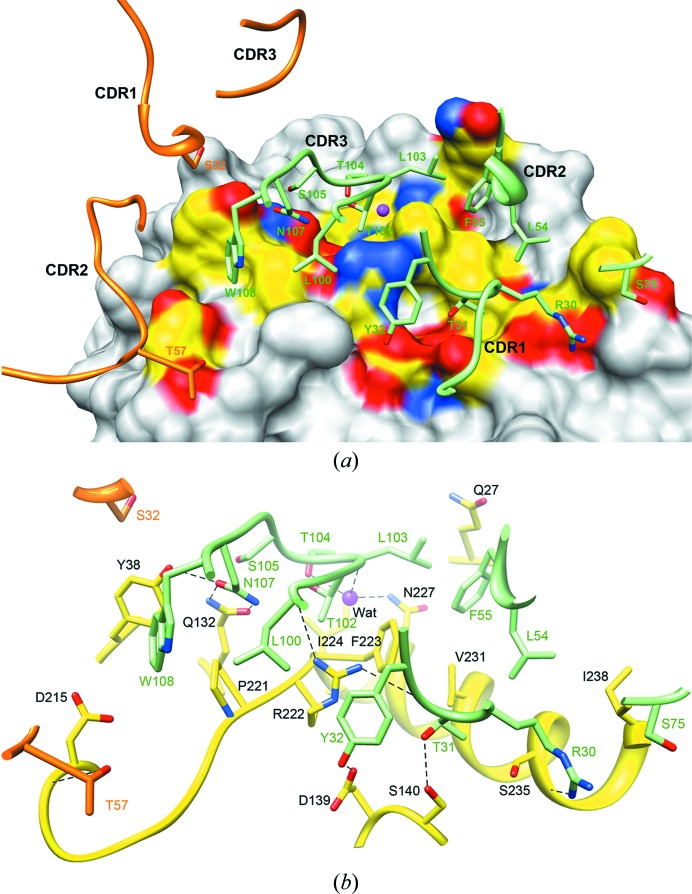
HSV-2 gD–E317-Fab interaction interface. (*a*) The epitope on HSV-2 gD is shown as an electrostatic surface that contains protrusions and concave cavities for water molecules and amino acids packed with the CDRs of E317-Fab. The heavy-chain and light-chain CDRs are shown as ribbons and the contacting residues are shown as stick models with green and orange C atoms. The water molecule is shown here as a magenta sphere. (*b*) The corresponding interactions are depicted using stick models. gD is shown as a yellow ribbon and sticks with yellow C atoms. All residues involved in direct contact between gD and E317-Fab are indicated and shown as sticks. Hydrogen bonds are shown as dashed lines. A water molecule (magenta) is also indicated.

Fig. 3 ▶ shows the interface interactions between HSV-2 gD and E317-Fab. The E317-binding site displays a conformational epitope located on the α3-helix external surface of gD. The central region is composed of a continuous linear peptide from residues 221 to 224 (PRFI) and extends to residues Asn227, Val231 and Ser235 on helix α3. The residues Gln27, Tyr38, Asp139, Ser140, Asp215 and Ile238 surround this central region to form a discontinuous conformational region for E317 recognition. The paratope on E317 consists of all three heavy-chain CDRs and two light-chain CDRs (CDR-L1 and CDR-L2). In the current structure, gD has a surface area of 11 240 Å^2^, of which an area of 2410 Å^2^ is buried by E317-Fab upon complex formation. A large area of the interface is buried by the heavy chain, which involves amino acids Gln27, Tyr38, Gln132, Asp139, Ser140 and a peptide from residues 221 to 238 in the discontinuous epitope, covering approximately 2020 Å^2^, or approximately 83% of the interface.

The side chains of residues Tyr32, Thr31 and Arg30 in the heavy-chain CDR-H1 make hydrogen bonds to the side chains of Asp139 and Ser140 and the backbone O atom of Ser235 of gD, respectively (Fig. 3 ▶ and Table 2 ▶). Two hydrophobic side chains in the heavy-chain CDR-H2, Leu54 and Phe55, insert into a hydrophobic cavity on gD and make nonpolar interactions with residues Gln27, Phe223, Val231 and Ile238 of gD. Arg222 is located at the epitope centre, with its NH1 and NH2 atoms hydrogen bonded to the backbone O atoms of Leu100 and Thr31 from CDR-H3 and CDR-H1, respectively. Furthermore, Gln132 and Tyr38 of gD make strong hydrogen-bond interactions with the Asn107 side chain of CDR-H3, and the hydrophobic Tyr108 of CDR-H3 stacks with Tyr38 and Pro221 of gD (Fig. 3 ▶ *b* and Table 2 ▶).

Interestingly, a water molecule was observed at the interface of gD and E317-Fab, with a lower *B* value (10.6 Å^2^) than average. It is hydrogen bonded to the backbone N atom of Leu103 and the side-chain OG1 atom of Thr104 in the heavy-chain CDR-H3 and the side-chain ND2 atom of gD Asn227 to mediate antigen–antibody complex formation, most likely as an important stabilizing element (Fig. 3 ▶ *b*). In addition, Asp215 and Tyr38 of gD contribute a small contact area with the light-chain variable fragment (Fv). The light-chain residue Ser32 in CDR-L1 is in nonpolar contact with Tyr38 in gD, and the OG1 atom of light-chain Thr57 in CDR-L2 is hydrogen bonded to the backbone O atom of Asp215 in gD (Fig. 3 ▶ and Table 2 ▶).

### 3.5. Sequence alignment

The gD sequences of HSV-1 and *Chimpanzee alpha-1 herpesvirus* (ChHV) were chosen for comparison with HSV-2 gD. Sequence alignment shows that HSV-2 gD has approximately 94.3% identity to ChHV gD over 368 residues and 84.8% identity to HSV-1 gD over 369 residues. The core domain and loop regions are highly conserved in these three different strains of virus (Fig. 2 ▶ *b*). The residues responsible for E317-Fab interaction in HSV-2 gD are exactly the same in ChHV gD, and only one residue (Gln27) is different in HSV-1 gD. In the HSV-2 gD–E317-Fab complex structure the side-chain protrusion of Gln27 does not provide any electrostatic interaction with E317; instead, it only has van der Waals interactions with Phe55 and Leu103 at distances of 3.97 and 3.78 Å, respectively (Table 2 ▶). The equivalent residue is an Arg in HSV-1 gD, which can provide a similar nonpolar interaction with E317.

### 3.6. Germline gene analysis

The human mAb E317 belongs to subclass IgG1 with a kappa (κ) light chain, and the closest homologous germline genes for mAb E317, as predicted by *IMGT/V-QUEST* and junction analysis (Lefranc *et al.*, 2012 ▶), are homsap IGHV1-69*04 F, homsap IGHJ5*02 F and homsap IGHD4-11*01 ORF in the heavy chain, and homsap IGKV3-20*01 F and homsap IGKJ4*01 F in the light chain. The positions of the CDRs according to the IMGT nomenclature are also predicted (Fig. 2 ▶ *c*). When aligned, the V_H_ and V_L_ amino-acid sequences for the respective germline sequences reveal that E317 has somatic hypermutations during antibody maturation, resulting in 27 amino-acid substitutions, among which 23 somatic mutations can be identified in the heavy chain, while the light chain contains the other four; thus, most somatic mutations are found in the heavy chain.

The structural data described above indicate that five somatic mutations (Arg30, Thr31, Leu54, Phe55 and Leu103) in the heavy chain participate in direct interaction with the antigen, and no substitution contributes to light-chain antigen interaction. Substitution with Arg30 leads to the formation of a hydrogen bond, and Thr31 provides an additional hydrophobic contact with gD. Interestingly, the side chains of three hydrophobic residues, Leu54, Phe55 and Leu103, form a hydrophobic protrusion that contacts gD (Fig. 3 ▶). They were mutated from smaller hydrophobic amino acids (Ile54, Leu55 and Val103) to larger side chains, thereby enhancing the hydrophobic interaction. The results demonstrate that somatic hypermutations in the heavy chain make a positive contribution to the affinity maturation of mAb E317, while those in the light chain do not contribute significantly to epitope binding.

### 3.7. Complex formation by analytical ultracentrifugation

HSV-2 gD and the Fab fragment of E317 form a 1:1 complex that can be isolated and crystallized. Based on the observed HSV-2 gD–E317-Fab complex structure, the epitopes in HSV-­2 gD are highly conserved in HSV-1 gD, except that Gln27 is replaced by an Arg in HSV-1 gD. To confirm the complex formation of both types of HSV gD with E317 in solution, purified Fab and gD were mixed in a 1:1 molar ratio and examined by sedimentation-velocity (SV) experiments to determine their complex formation.

**Figure 4:**
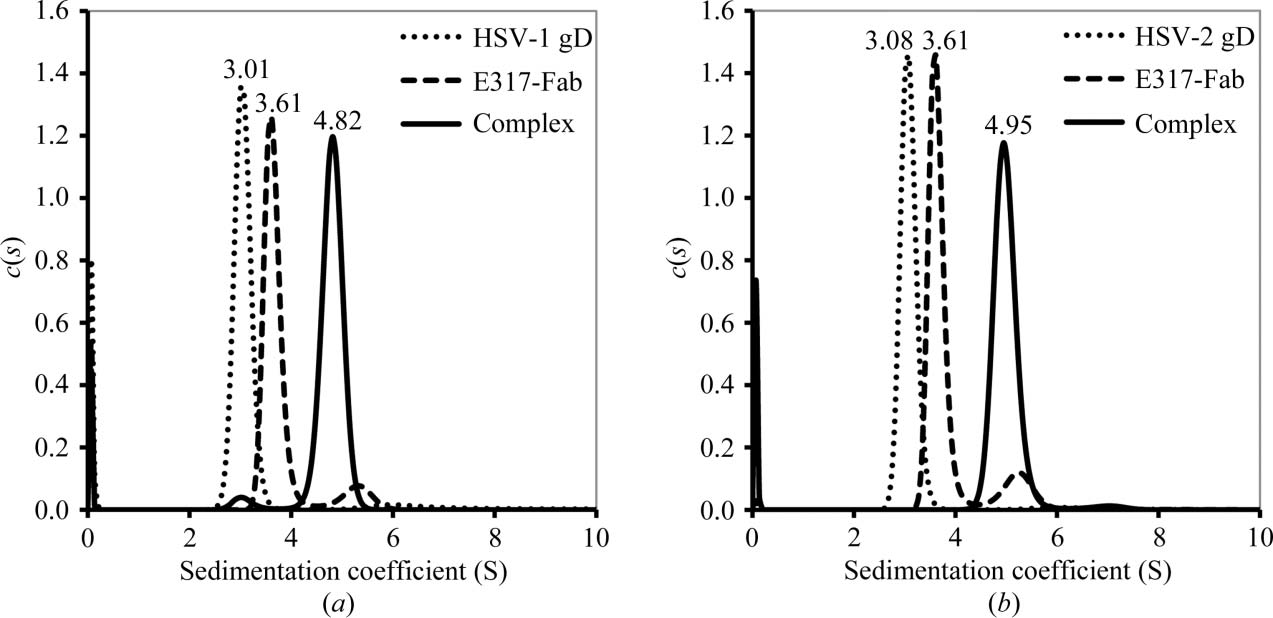
gD–E317-Fab complex formation. (*a*) The sedimentation-coefficient distribution profiles of HSV-1 gD (dotted line), E317-Fab (dashed line) and the gD–E317-Fab complex (solid line). (*b*) The sedimentation-coefficient distribution profiles of HSV-2 gD, E317-Fab and their complex.

The continuous *c*(*s*) distribution indicates that E317-Fab and HSV-2 gD have sedimentation coefficients (*S*) of 3.61 and 3.08S and calculated masses of 46.0 and 42.3 kDa, respectively. The associated complex has an *S* value of 4.95S (84.4 kDa), and the single peak also indicates that the complex has a very low disassociation rate owing to large energy barriers (Fig. 4 ▶ *a* and Table 3 ▶). The *S* values of HSV-1 gD and the E317-Fab–HSV-1 gD complex are 3.01 and 4.82S, with calculated masses of 41.5 and 79.9 kDa, respectively, corresponding to complex formation of E317-Fab and HSV-2 gD (Fig. 4 ▶ *b* and Table 3 ▶). These results demonstrate the ability of E317-Fab to associate with HSV-1 gD in solution.

### 3.8. Comparison of the interactions of E317-Fab and nectin-1 with gD

**Figure 5:**
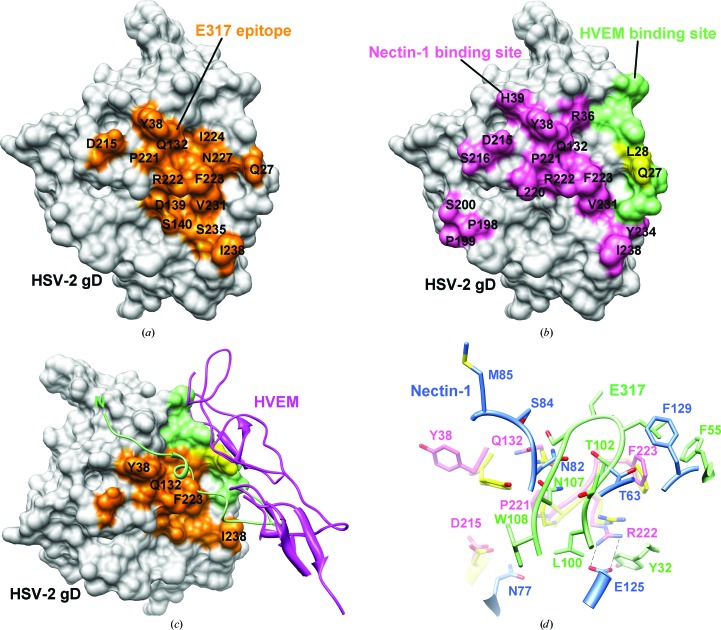
Comparison of E317 epitope and receptor-binding sites. (*a*) Surface representation of HSV-2 gD with residues involved in E317-Fab binding highlighted in orange. (*b*) The receptor-binding sites on HSV-1 gD corresponding to HSV-2 gD are highlighted. The HVEM-binding sites are shown in green and yellow (residues 25–32). The residues involved in nectin-1 binding are coloured pink and yellow. Both Gln27 and Leu28 make contact with two receptors. (*c*) The HSV-1 gD–HVEM complex structure (PDB entry 1jma) was superimposed onto the HSV-2 gD structure; the N-terminal region of HSV-1 gD and HVEM are shown as green and magenta ribbons, respectively. E317 epitopes and HVEM-binding sites are shown as in the above figures. (*d*) Comparison with the interactions in gD–nectin-1. The key contact residues in the gD–nectin-1 interface are displayed as sticks and are labelled. The C atoms of HSV-­1 gD and nectin-1 are coloured pink and blue, respectively. E317 and HSV-2 gD are shown as in Fig. 3 ▶(*b*).

In the structure of gD–E317-Fab, E317 binds to a similar site but adopts a completely different binding mode from that in the gD–nectin-1 complex. The gD residues Tyr38, Gln132, Asp215, Pro221, Arg222 and Phe223, which are involved in binding nectin-1, also participate in interactions with mAb E317 (Figs. 5 ▶ *a* and 5 ▶ *b*). Fig. 5 ▶(*d*) shows a comparison of the gD interactions with E317 and with nectin-1. In the gD–nectin-1 structure, gD residues Arg222 and Phe223 provide strong interactions with nectin-1. Nectin-1 residue Glu125 forms an intermolecular salt bridge with gD residue Arg222, which is hydrogen-bonded to the E317 CDR-H1 Thr31 and CDR-H3 Leu100 backbone O atoms in the gD–E317-Fab structure. The phenyl ring of gD residue Phe223 has a similar orientation in both complex structures in spite of the different interactions. It is in hydrophobic contact with Thr63 in nectin-1 and by T-­shaped π-stacking with the phenyl group of Phe129. However, the gD Phe223 phenyl ring has a different stacking mode with the Phe55 phenyl group of E317 CDR-H2, and it also forms a hydrophobic sandwich with Thr102 of E317 CDR-H3. In addition, E317 CDR-H3 residue Asn107 has a van der Waals interaction with gD Pro221 and causes the side chains of Tyr38 and Gln132 of gD to rotate and form a polar network. The side chain of Tyr38 rotates by ∼90° not only to accommodate hydrogen bonds to Asn107 but also to provide a stacking force with Trp108 of the E317 CDR-H3 loop (Fig. 5 ▶ *d*).

### 3.9. Neutralization mechanism

The binding of gD to targets such as HVEM and nectin-1 is required for HSV entry into host cells. HVEM-binding and nectin-1-binding sites on gD have been identified previously (Connolly *et al.*, 2005 ▶, 2011 ▶; Carfí *et al.*, 2001 ▶; Whitbeck *et al.*, 2001 ▶), and the crystal structures of gD–HVEM and gD–nectin-1 complexes also provide strong evidence for gD–receptor interactions (Carfí *et al.*, 2001 ▶; Di Giovine *et al.*, 2011 ▶). In the gD–HVEM structure, the flexible N-terminus of gD folds into a hairpin conformation upon HVEM binding (Carfí *et al.*, 2001 ▶). Fig. 5 ▶(*c*) shows a comparison of E317-binding and HVEM-binding sites on gD, in which the N-­terminal 1–18 region of gD occupies the space near or within the mAb E317 and nectin-1-binding sites, such as Tyr38, Gln132 and Phe223. This N-terminal region is conformationally flexible in unbound gD and its presence does not interfere with recognition by mAb E317 and nectin-1. Moreover, the two receptors have overlapping binding areas and residues (Leu28 and Gln27) on gD. The recent structure of the complex of gD and nectin-1 showed that most of the nectin-1-binding residues in gD differ from the HVEM-binding residues and that the formation of the N-terminal hairpin is not required for nectin-­1 binding, whereas Arg222 and Phe223 in gD play important roles in the intramolecular interaction with nectin-1 (Di Giovine *et al.*, 2011). A comparison of the mAb E317 and the nectin-1 binding sites on gD shows that the overlapping binding residues comprise Tyr38, Gln132, Asp215, Pro221, Arg222, Phe223, Val231 and Ile238, indicating that mAb E317 and nectin-1 have competitive interactions with gD. For HSV neutralization, mAb E317 binding to gD can block both interactions with HVEM and nectin-1 to prevent HSV entry into host cells (Lai & Chan, 2012 ▶).

## 4. Discussion

To understand how human mAb E317 specifically recognizes HSV gD and neutralizes the viral infection, crystal structures of the HSV-specific mAb E317 Fab fragment and the complex of the Fab with HSV-2 gD were determined in this study. Additionally, the complex crystal also reveals the first structure of HSV-2 gD. These structures provide a molecular basis for understanding the interactions between gD and its neutralization antibody E317. Approximately 83% of the interaction surface buried on the Fab is located on the heavy chain, with hydrogen-bond interactions and large hydrophobic contact regions. Importantly, the epitopes for mAb E317 were identified and they suggest a possible mechanism of how E317 blocks the two receptors HVEM and nectin-1 from interacting with gD for HSV cell entry.

The overall structure of HSV-2 gD has an Ig-like domain with a long insertion between strands *G* and 4. Two glycosylation sites can also be observed in the crystal structure. The glycan on Asn121 forms a hydrogen bond with Gln41, and it is believed to help with the contacts between loop α1′C and loop 2A′ to stabilize loop 2A′. The region between strands *G* and 4 connects to the core domain, with two disulfide bridges between Cys66 and Cys189 and between Cys106 and Cys202, and thus provides a folded conformation for its N-­terminal and C-­terminal extensions to contact and bind the receptor. In other known structures of HSV-1 gD, the N-­terminal extension was observed in the gD285–HVEM complex and the C-­terminal extension was observed in an N-­terminally truncated gD(23–306)_307C_ structure that had an extra cysteine mutation at the C-terminus (Carfí *et al.*, 2001 ▶; Krummenacher *et al.*, 2005 ▶). These observations indicate that both terminal extensions are highly dynamic.

In the gD–E317-Fab complex structure described here, the terminal regions of gD cannot be observed. This is consistent with previous studies and indicates that the existence of these flexible regions does not affect the binding of E317 to gD. The formation of the N-terminal hairpin facilitates the binding of HVEM to gD. The gD285–HVEM complex structure was superimposed on the gD of the gD–E317-Fab complex to compare the spatial overlap and epitope occupancy of HVEM and E317. The N-terminal region is located on a surface groove and overlaps with Phe223 of HSV-2 gD in the gD–E317-Fab complex structure. The residues Gln27, Tyr38, Gln132, Phe223, Ile224, Asn227 and Ile238 of gD involved in E317 binding are also involved in the interaction between its N-terminus and HVEM (Fig. 5 ▶ *c*). Therefore, the presence of the bound mAb E317 should block the binding groove of the gD N-terminus to affect the formation of the hairpin in contact with HVEM. In previous studies, Tyr38, Asp215, Arg222 and Phe223 on gD were identified as major neutralizing antigenic sites (Whitbeck *et al.*, 1999 ▶) whose functions are to interact with nectin-1 (Connolly *et al.*, 2011 ▶; Di Giovine *et al.*, 2011 ▶). That E317 binds to a conformational epitope which comprises residues Tyr38, Asp215 and Arg222 has also been demonstrated in a patent (Lai & Chan, 2012 ▶).

Comparison of the binding sites for E317 and nectin-1 on gD shows a large overlap region. It is evident that E317 has spatial overlap and competes with nectin-1 to interact with gD. In the gD–E317-Fab complex a total of 2410 Å^2^ of the surface area of gD is buried by E317-Fab. This interacting area is nearly threefold larger than the nectin-1 binding area (836 Å^2^) in the gD–nectin-1 complex structure (Di Giovine *et al.*, 2011 ▶). All identified neutralizing antigenic sites appear to be blocked by E317, preventing the binding of nectin-1, in which it is presumed that the heavy-chain CDRs are dominant and the light-chain CDRs play an auxiliary role. Furthermore, among the contributions of CDRs to gD recognition, the CDR-H3 loop accounts for a large proportion of the total buried surface area (Fig. 3 ▶ *a*). The structure of CDR-H3 shows a similar loop conformation (Fig. 1 ▶ *d*) in the bound and unbound structures and it makes specific contacts with the neutralizing antigenic sites (Tyr38, Arg222 and Phe223; Table 2 ▶), suggesting the requirement of CDR-H3 for viral neutralization and indicating the important role of the CDR-H3 structure in its antigen-binding function.

The analytical ultracentrifugation results indicated that E317-Fab not only forms a complex with HSV-2 gD but also associates with HSV-1 gD in solution. The single-chain Fv E317 significantly inhibits HSV-1 (strain KOS) and HSV-2 (strain 186) replication in Vero cells, with IC_50_ values of 5.65 and 3.6 n*M*, respectively (Lai & Chan, 2012 ▶). The location of the E317 epitope may explain the slight difference between these two HSV strains. The epitope is highly conserved in both human HSV strains; only a Q27R mutation was found, which resulted in a 1.6-fold decrease in its inhibitory activity in HSV-­1.

Viral glycoproteins that are required for virus–cell fusion during entry into host cells have become potential targets for antiviral vaccine and neutralizing antibody development. The broadly neutralizing antibodies tend to recognize the receptor-binding site and membrane-fusion machinery (Julien *et al.*, 2012 ▶). However, the dense shield by oligosaccharides and the resistant variants make it difficult to generate effective broadly neutralizing antibodies against, for example, influenza haemagglutinin (HA) and human immunodeficiency virus-1 (HIV-1) envelope protein (Env) (Clementi *et al.*, 2012 ▶; Huang *et al.*, 2012 ▶).

A recent study suggests that glycoprotein D bound to receptor can associate with both gH/gL and gB to drive HSV fusion (Atanasiu *et al.*, 2010 ▶). For anti-HSV therapies, the antibody or inhibitor can be designed to disrupt the formation of fusogen, and some monoclonal antibodies and peptides have been focused on blocking gH/gL activity (Nicola *et al.*, 1998 ▶; Tang *et al.*, 2011 ▶), such as neutralizating antibody 52S, LP11 and the gp42 peptides (Chowdary *et al.*, 2010 ▶; Kirschner *et al.*, 2007 ▶). It is well established that gD can induce monoclonal antibodies to block the interaction between gD and HVEM for viral neutralization (Nicola *et al.*, 1998 ▶). The crystal structures of gD–receptor complexes indicate that nectin-1 and HVEM recognize different sites on gD. As a challenge to produce a monoclonal antibody that can block both receptors, the specific and high-affinity monoclonal antibody E317 was produced to complex with HSV-2 gD in this study. The E317-binding site was mapped as a conformational epitope which has large overlap regions with the nectin-1-binding site. The binding of E317 to gD not only blocks the binding of nectin-1, but also interferes with the N-terminal hairpin formation to neutralize HVEM-mediated entry. Although neutralization can depend on aggregation by cross-linking with intact multi-valent antibodies, and receptor blocking is not necessarily the primary mechanism of neutralization, this gD–E317-Fab complex can nonetheless provide a structural basis for affinity optimization, while the conserved epitope can serve as a template for HSV neutralization and will probably find applications in gD-based HSV vaccine design. The results further strengthen the previously demonstrated therapeutic and diagnostic potential of the monoclonal antibody E317.

## Supplementary Material

PDB reference: E317-Fab, 3w9d

PDB reference: gD–E317-Fab complex, 3w9e

Supplementary material file. DOI: 10.1107/S0907444913016776/mv5090sup1.pdf

## Figures and Tables

**Table 1:** Data-collection and refinement statistics Values in parentheses are for the highest resolution shell.

	E317-Fab	HSV-2 gD–E317-Fab
Data collection		
Wavelength (Å)	1.5418	0.9000
Space group	*P*2_1_2_1_2_1_	*P*2_1_2_1_2_1_
Unit-cell parameters (Å)	*a* = 81.78, *b* = 90.72, *c* = 128.20	*a* = 69.03, *b* = 91.09, *c* = 141.54
Resolution (Å)	30–2.32 (2.40–2.32)	30–2.30 (2.38–2.30)
Observed reflections	132310	256296
Unique reflections	40644	40198
*R*_merge_ (%)	5.5 (33.8)	6.1 (48.9)
〈*I*/σ(*I*)〉	29.3 (5.7)	26.2 (4.6)
Completeness (%)	96.3 (96.3)	99.0 (97.7)
Multiplicity	3.3 (3.1)	6.4 (6.3)
Refinement		
Resolution (Å)	30–2.32	30–2.30
No. of reflections (*R*_work_/*R*_free_)	36995/1955	37832/1891
*R*_work_/*R*_free_ (%)	20.8/26.6	20.3/25.5
No. of atoms		
Protein	6412	5043
Glycan		64
Water	557	253
Average *B* factor (Å^2^)		
Protein	30.54	52.6
Glycan		85.3
Water	33.56	48.0
R.m.s.d.		
Bond lengths (Å)	0.007	0.008
Bond angles (°)	1.54	1.35
Ramachandran statistics† (%)		
Most favoured	86.1	90.5
Additionally allowed	12.9	9.0
Generously allowed	0.4	0.0
Disallowed	0.6	0.5

† The stereochemistry of the model was validated using *PROCHECK*.

**Table 2:** HSV-2 gD–Fab direct interactions The distance cutoff used for hydrogen bonds is 3.4 Å, while 4.0 Å is used for nonpolar interactions.

gD	Fab heavy chain	Fab light chain	Distance (Å)
Hydrogen bonds			
Tyr38 OH	Asn107 OD1		2.85
Gln132 NE2	Asn107 OD1		2.84
Gln132 OE1	Asn107 ND2		3.31
Asp139 OD2	Tyr32 OH		2.35
Ser140 OG	Thr31 OG1		2.81
Asp215 O		Thr57 OG1	2.78
Arg222 NH1	Leu100 O		2.99
Arg222 NH2	Thr31 O		2.84
Ser235 O	Arg30 NH1		2.90
Nonpolar (distance corresponds to the closest atom pair)			
Gln27	Phe55		3.97
Gln27	Leu103		3.78
Tyr38	Trp108		3.48
Tyr38		Ser32	3.98
Gln132	Ser105		3.41
Ser140	Thr31		3.87
Pro221	Trp108		3.73
Arg222	Tyr32		3.35
Arg222	Thr102		3.33
Phe223	Phe55		3.83
Phe223	Thr102		3.58
Ile224	Thr104		3.92
Asn227	Leu103		3.94
Val231	Thr31		3.73
Val231	Phe55		3.46
Ser235	Arg30		3.78
Ser235	Thr31		3.61
Ile238	Leu54		3.64
Ile238	Ser75		3.55

**Table 3:** Sedimentation-coefficient results and molecular weights

	HSV-1 gD	HSV-2 gD	E317-Fab	HSV-1 gD–E317-Fab	HSV-2 gD–E317-Fab
*S* (S)	3.01	3.08	3.61	4.82	4.95
MW† (kDa)	41.5	42.3	46.0	79.9	84.4

† The molecular weights were calculated using sedimentation-velocity experiments.

## Footnotes

1 Supplementary material has been deposited in the IUCr electronic archive (Reference: MV5090). Services for accessing this material are described at the back of the journal.
