# Supplementary material for: Structural basis for the antibody neutralization of Herpes simplex virus
Source: Acta Crystallogr D Biol Crystallogr. 2013 Sep 20;69(Pt 10):1935–45. doi: 10.1107/S0907444913016776 (PMC3792640; doi:10.1107/S0907444913016776)
Supplement: Supplementary file 1 [file d-69-01935-sup1.pdf]

Supplementary Material

Supplementary Figure S1. The variable fragments of mAb E317 are numbered according to the kabat numbering scheme (http://www.bioinf.org.uk/abysis). The CDR positions are also predicted.

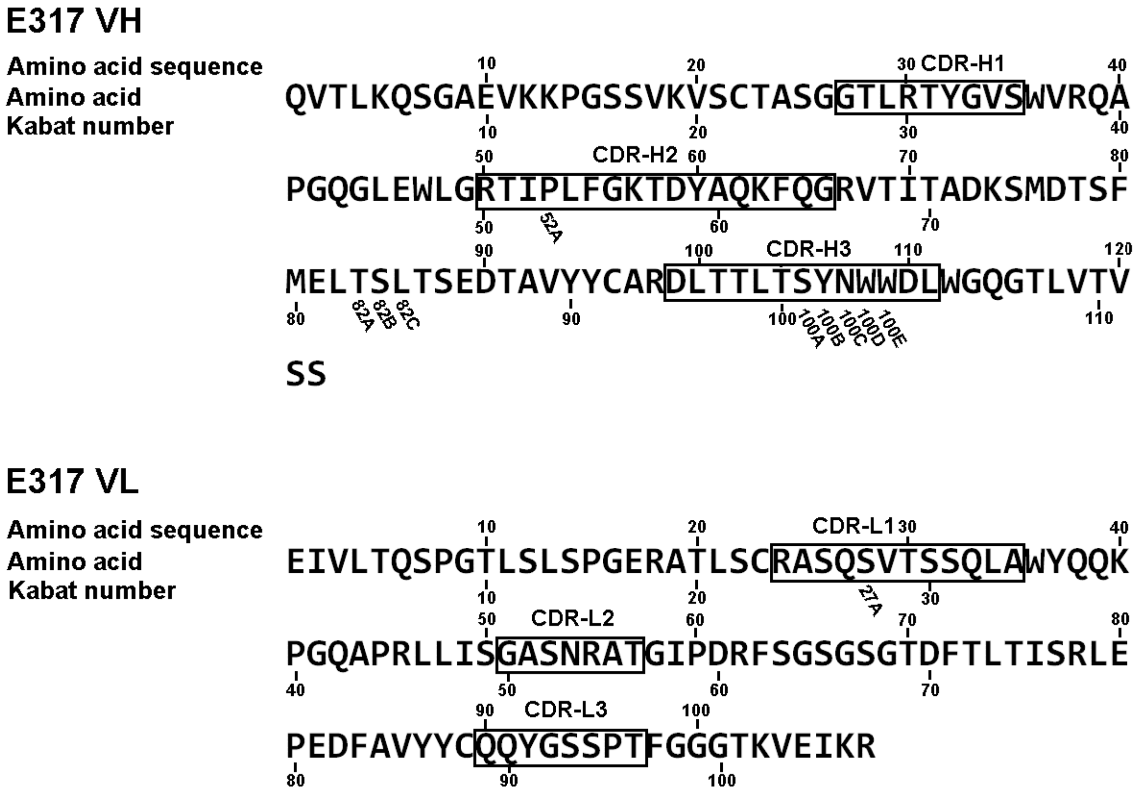

Supplementary Table S1. The rmsd values of 18 Cα atoms pairs in the CDRs.

	CDR-H1	CDR-H2	CDR-H3	CDR-L1	CDR-L2	CDR-L3
Free Fab HL / gD-bound Fab	0.40	1.04	0.30	0.19	0.03	0.17
Free Fab H'L' / gD-bound Fab	0.78	0.29	0.33	0.78	0.03	0.24
Free Fab HL / Free Fab H'L'	0.90	1.10	0.21	0.78	0.01	0.11
